# Quality of comprehensive assessment among severely ill TB patients referred after triaging in southern India

**DOI:** 10.5588/pha.23.0051

**Published:** 2024-03-01

**Authors:** H. D. Shewade, A. Frederick, S. Kiran Pradeep, T. Daniel Rajasekar, G. Kiruthika, T. Bhatnagar, K. V. Suma, P. Ravichandran, K. Gayathri, R. Vijayaprabha, D. P. Pathinathan, D. Chidambaram, K. Sivagami, R. K. Janani, T. S. Selvavinayagam, R. Ramachandran, M. V. Murhekar

**Affiliations:** ^1^ ICMR–National Institute of Epidemiology (ICMR-NIE), Chennai,; ^2^ State TB Cell, Government of Tamil Nadu, Chennai,; ^3^ Office of the WHO Representative to India, WHO Country Office, New Delhi,; ^4^ Directorate of Public Health and Preventive Medicine, Government of Tamil Nadu, Chennai, India

**Keywords:** TNKET, severe TB, clinical assessment, India, assessment quality, operational research

## Abstract

To reduce TB deaths, Tamil Nadu, a southern Indian state, implemented the first state-wide differentiated TB care strategy starting April 2022. Triage-positive severely ill patients are prioritised for comprehensive assessment and inpatient care. Routine program data during October–December 2022 revealed that documentation of total score after comprehensive assessment was available in only 39%, possibly indicating poor quality of comprehensive assessment. We confirmed this using operational research. The case record form to record comprehensive assessment was used only in 26% and among these, the completeness and correctness in filling out the form were sub-optimal. There is a clear need to enhance the quality of comprehensive assessments.

## Abstract (fr)

Depuis avril 2022, le Tamil Nadu, un État du sud de l'Inde, a mis en œuvre la première stratégie de soins différenciés pour la TB à l'échelle de l'État afin de réduire le nombre de décès dus à la TB. Les personnes gravement malades ayant obtenu un résultat positif au triage sont prioritaires pour une évaluation complète et des soins hospitaliers. Les données du programme de routine entre octobre et décembre 2022 ont révélé que la documentation du score total après l'évaluation complète n'était disponible que dans 39% des cas, ce qui pourrait indiquer une mauvaise qualité de l'évaluation complète. Nous l'avons confirmé par le biais d'une recherche opérationnelle. Le formulaire de dossier pour enregistrer l'évaluation complète n'a été utilisé que dans 26% des cas et, parmi ceux-ci, l'exhaustivité et l'exactitude du remplissage du formulaire n'étaient pas optimales. Il est manifestement nécessaire d'améliorer la qualité de l'évaluation complète.

In 2020–2021 during the COVID-19 pandemic, there was an increase in estimated TB deaths and the reported TB death rate in India, including Tamil Nadu.^1,2^ Tamil Nadu is a southern Indian state with 70 million population and approximately 0.1 million TB notifications per year.^1^ With the aim to reduce the TB death rate, Tamil Nadu implemented the first state-wide (excluding Chennai, the capital city) differentiated TB care strategy among all adult (≥15 years) TB patients notified from public health facilities (not known to be drug-resistant at diagnosis) in April 2022.^3–5^ The strategy is called Tamil Nadu *Kasanoi Erappila Thittam*, (TNKET) meaning Tamil Nadu TB death-free project in Tamil.^3,4^

Using a paper-based triage tool, all adults with TB are triaged for severe illness at diagnosis.^3–5^ Those detected with very severe undernutrition (body mass index ≤14 kg/m^2^ or 14.1–16 kg/m^2^ with pedal oedema) or respiratory insufficiency (respiratory rate more than 24 per min or oxygen saturation <94%) or poor performance status (unable to stand without support), defined as ‘triage-positive’, are identified and prioritised for referral to a nodal inpatient care facility for comprehensive assessment (using paper-based case record form [CRF]) and inpatient care using an inpatient care guide for people with TB with severe illness (for details on this, the 2-page TNKET standard operating procedure containing links to the paper-based triage tool, the CRF, inpatient care guide, and details about the monitoring and evaluation framework, see Supplementary Data, at https://doi.org/10.6084/m9.figshare.24564403; all Supplementary Data are available under the terms of the Creative Commons Attribution 4.0 International License [CC BY 4.0]).^3–5^ The key variables required for monitoring the TNKET care cascade were transcribed from paper-based tools to Severe TB Web Application (TB SeWA) by the Senior Treatment Supervisor (STS; sub-district-level staff in charge of TB patients in a population of approximately 50–100,000; for details see Supplementary Figure S1 at https://doi.org/10.6084/m9.figshare.24625026.v2).^3–5^

The CRF is a single paper tool with front and back print. The initial section contains information about the facility where the patient was diagnosed and the contact details of STS, has to be filled out by the referring STS. Details regarding clinical, laboratory and radiological assessment are subsequently filled out by the nodal physician at the designated inpatient care facility. Triage-positive patients are scored based on a set of 16 indicators.^3^ Those with a total score of more than one or presence of emergency criteria are confirmed as severely ill.^3,4,6^ Unless there is an error in triaging at diagnosis, triage-positives get confirmed as ‘severely ill’. Those with a total score of more than three or with very severe undernutrition are recommended admission to a facility with high dependency and/or an intensive care unit of the teaching hospital.^3,4^ This is followed by documentation of issues that need to be addressed during inpatient care. The patient is discharged after fulfilling the discharge criteria; date of admission, discharge and admission outcome are documented.

The paper-based CRF was introduced in August 2022. In addition to the ongoing transcription of comprehensive assessment (yes/no) in TB SeWA, we added the transcription of total scores after comprehensive assessment from August 2022.

## ASPECTS OF INTEREST

By the end of 2022, nearly 80% of triage-positive were identified, comprehensively assessed and admitted within a median of 1 day of diagnosis.^5^ The average admission duration was 6 days.^5^ This, along with quality comprehensive assessment and inpatient care, will increase the likelihood of TNKET achieving its goal of reducing TB death rate.^3,4^

Although comprehensive assessment among triage-positive was documented as ‘yes’ in TB SeWA for 90% patients, the total score was documented in only 39% in the quarter following the introduction of the CRF (October–December 2022), possibly indicating poor quality of comprehensive assessment. To investigate this, we conducted operational research in 10 (out of 30) randomly selected TB programme districts to evaluate the quality of the comprehensive assessment conducted in terms of completeness and accuracy in filling out the CRF.

Of the 4,058 TB patients notified, 446 were triage-positive and of these, 402 underwent comprehensive assessment (‘yes’ in TB SeWA as on 10 March 2023; see Supplementary Figure S2 at https://doi.org/10.6084/m9.figshare.24625026.v2). During March–May 2023, we visited the 10 study districts to obtain a copy of the filled CRF. Of the 10 districts, only four utilised the CRF. Of these 402 patients, the CRF was filled out for 104 (25.9%) patients. We assessed the completeness and correctness of these 104 CRFs.

The seven fields meant for the STS before referral were complete in 29 forms (27.9%). Complete documentation of clinical, radiological and laboratory assessment was documented in 19 forms (18.3%). Among the six questions related to undernutrition assessment, 32 forms (30.8%) had all six responses.

Of the 104 forms that had been filled out, 54 (51.9%) contained scores for each of the 16 indicators, along with a documented total score. Only four forms (3.8%) had documented confirmation of severe illness, whether or not high-dependency unit care was required, as well as an ‘issue to be addressed during inpatient care’. A total of 84 forms (80.8%) had all fields related to admission and discharge-related details filled out. A comprehensive breakdown of the completeness of documentation in the CRF is provided in Tables 1 and 2.

**TABLE 1.** Completeness of paper-based TNKET CRF among triage-positive adults with TB who were documented to be comprehensively assessed, Tamil Nadu, India, October–December 2022 (*n* = 104).*

	Total
Key indicator	*n* (%)
All seven fields to be written by the senior treatment supervisor were filled	29 (27.9)
All three general fields to be filled out by the nodal physician were completed	90 (86.5)
All undernutrition assessment fields were filled	32 (30.8)
All basic investigations were documented	19 (18.3)
All 16 comprehensive assessment scores were documented	54 (51.9)
All confirmation of severe illness-related fields were filled	4 (3.8)
All three fields related to admission and discharge were filled	84 (80.8)
Number of completely filled out CRFs	0 (0.0)

* 104 of 402 triage-positive adults with TB who were documented as comprehensively assessed in severe TB web application; 104 had the CRF filled at the nodal inpatient care facility.

TNKET = Tamil Nadu *Kasanoi Erappila Thittam* (meaning Tamil Nadu TB death-free project in Tamil); CRF = case record form; STS = senior treatment supervisor.

**TABLE 2.** Completeness of paper-based TNKET CRF among triage-positive adults with TB that were documented to be comprehensively assessed, Tamil Nadu, India, October–December 2022 (*n* = 104).*

Indicator	*n* (%)
Details to be filled out by the senior treatment supervisor	
Number of CRFs with the patient’s *Ni-kshay* identifier (unique ID)	92 (88.5)
Number of CRFs with the contact details of the senior treatment supervisor	54 (51.9)
Details to be filled by the nodal physician	
General details	
Number of CRFs with the date of comprehensive assessment	93 (89.4)
Number of CRFs with the name and details of the nodal facility filled out	93 (89.4)
Number of CRFs with the name and details of the nodal physician filled out	91 (87.5)
History	
Number of CRFs with completed history of haemoptysis	56 (53.8)
Number of CRFs with completed history of substance use	49 (47.1)
Number of CRFs with completed history of chronic illness	56 (53.8)
Assessment of undernutrition status	
Number of CRFs with documented general condition	57 (54.8)
Number of CRFs with documented weight	94 (90.4)
Number of CRFs with documented height	94 (90.4)
Number of CRFs with documented BMI or MUAC measures	98 (94.2)
Number of CRFs with documented severe undernutrition status	88 (84.6)
Number of CRFs with documented very severe undernutrition status	61 (58.6)
Assessment of vital signs	
Number of CRFs with documented pulse rates	89 (85.6)
Number of CRFs with documented temperatures	64 (61.5)
Number of CRFs with documented respiratory rates	95 (91.3)
Number of CRFs with documented oxygen saturation rates	103 (99.0)
Number of CRFs with documented icterus	89 (85.6)
Number of CRFs with documented pedal oedema status	91 (87.5)
Number of CRFs with documented blood pressure measures	101 (97.1)
Radiological and laboratory investigations	
Number of CRFs with documented chest radiograph findings	43 (41.3)
Number of CRFs with documented liver function test scores	48 (46.2)
Number of CRFs with documented renal function test scores	47 (45.2)
Number of CRFs with documented haemoglobin levels	64 (61.5)
Number of CRFs with documented WBC count	60 (57.7)
Number of CRFs with documented HIV and ART status	101 (97.1)
Number of CRFs with documented random blood sugar levels	98 (94.2)
Scoring and confirmation of severe illness based on the comprehensive assessment	
Number of CRFs with documented pulse rates	76 (73.1)
Number of CRFs with documented BMI/MUAC measures	76 (73.8)
Number of CRFs with documented temperatures	71 (68.3)
Number of CRFs with documented blood pressure levels	69 (66.3)
Number of CRFs with documented respiratory rates	79 (76.0)
Number of CRFs with documented oxygen saturation rates	62 (59.6)
Number of CRFs with documented haemoglobin levels	63 (60.6)
Number of CRFs with documented icterus scores	60 (57.7)
Number of CRFs with documented pedal oedema scores	62 (59.6)
Number of CRFs with documented general condition scores	70 (67.3)
Number of CRFs with documented HIV scores	63 (60.6)
Number of CRFs with documented random blood sugar levels	68 (65.4)
Number of CRFs with documented WBC scores	66 (63.5)
Number of CRFs with documented chest radiograph scores	80 (76.9)
Number of CRFs with documented haemoptysis scores	64 (61.5)
Number of CRFs with documented total scores	97 (93.3)
Confirmation of illness and action taken	
Number of CRFs with documented confirmed severe illness status	43 (41.3)
Number of CRFs with documented HDU or ICU care requirement status	50 (48.1)
Number of CRFs with documented reasons for severe illness (at least one)	56 (53.8)
Admission details	
Number of CRFs with documented admission dates	95 (91.3)
Number of CRFs with documented discharge dates	95 (91.3)
Number of CRFs with a documented outcome of admission	86 (82.7)

* 104 of 402 triage-positive adults with TB who were documented as comprehensively assessed in severe TB web application; 104 had the CRF filled at the nodal inpatient care facility.

TNKET = Tamil Nadu *Kasanoi Erappila Thittam* (meaning Tamil Nadu TB death-free project in Tamil language); CRF = case record form; BMI = body mass index; MUAC = mid-upper arm circumference; WBC = white blood cell; ART = antiretroviral treatment; HDU = high-dependency unit; ICU = intensive care unit.

Of the 104 CRFs, we were able to objectively verify correctness of two variables: ‘severe’ and ‘very severe’ undernutrition status was correctly documented in 32 (30.8%); and in 12 forms (12.4%), the total score was correctly calculated.

## DISCUSSION

During October–December 2022, the failure to utilise the CRF seemed to be the cause behind the non-transcription of the total score (after comprehensive assessment) in TB SeWA. Even among those using the CRF, quality (completeness and accuracy) was sub-optimal. This has major implications in that it can potentially affect patient management adversely, resulting in missed opportunities for optimal care.

A possible reason might be the lack of awareness of the utility of the TNKET CRF among the nodal physicians. There is a clear need to intensify efforts to enhance the quality of the comprehensive assessment through reorientation of the district TB officers and nodal physicians. This is being done during the quarterly supportive supervisory visit to a district and online reorientation session. The transcription of the total scores in TB SeWA has increased to 79% in April–June 2023 from 39% in October–December 2022. Districts that initially did not use these forms have started implementing them. We are hopeful of a corresponding improvement in the quality of comprehensive assessment, which needs to be confirmed through a repeat assessment of completeness and correctness. This is essential if we are to move closer to the UN Sustainable Development Goal target of reducing TB deaths by 90% in 2030 compared to 2015.^7^
